# Full Body-Worn Textile-Integrated Nanomaterials and Soft Electronics for Real-Time Continuous Motion Recognition Using Cloud Computing

**DOI:** 10.1021/acsami.4c17369

**Published:** 2025-01-24

**Authors:** Kangkyu Kwon, Yoon Jae Lee, Suyeong Chung, Jimin Lee, Yewon Na, Youngjin Kwon, Beomjune Shin, Allison Bateman, Jaeho Lee, Matthew Guess, Jung Woo Sohn, Jinwoo Lee, Woon-Hong Yeo

**Affiliations:** † School of Electrical and Computer Engineering, Georgia Institute of Technology, Atlanta, Georgia 30332, United States; ‡ Center for Wearable Intelligent Systems and Healthcare, Georgia Institute of Technology, Atlanta, Georgia 30332, United States; § Department of Aeronautics, Department of Mechanical and Electronic Convergence Engineering, Kumoh National Institute of Technology, Gumi 39177, Republic of Korea; ∥ George W. Woodruff School of Mechanical Engineering, Georgia Institute of Technology, Atlanta, Georgia 30332, United States; ⊥ School of Mechanical System Engineering, Kumoh National Institute of Technology, Gumi 39177, Republic of Korea; # Department of Mechanical, Robotics, and Energy Engineering, Dongguk University, Seoul 04620, Republic of Korea; ¶ Wallace H. Coulter Department of Biomedical Engineering, Parker H. Petit Institute for Bioengineering and Biosciences, Neural Engineering Center, Institute for Materials, Institute for Robotics and Intelligent Machines, Georgia Institute of Technology, Atlanta, Georgia 30332, United States

**Keywords:** wearable electronics, textile-integrated sensors, cloud computing, motion recognition, deep learning

## Abstract

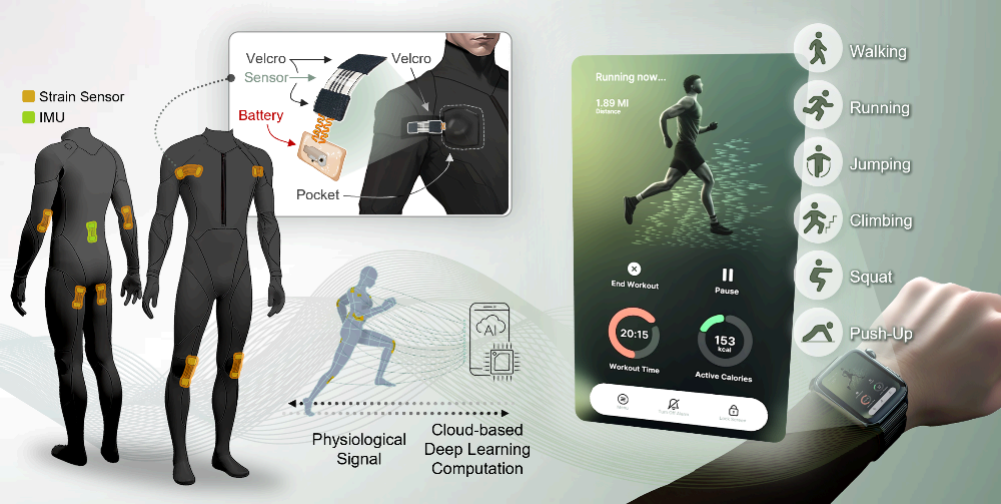

Recognizing human body motions opens possibilities for real-time observation of users’ daily activities, revolutionizing continuous human healthcare and rehabilitation. While some wearable sensors show their capabilities in detecting movements, no prior work could detect full-body motions with wireless devices. Here, we introduce a soft electronic textile-integrated system, including nanomaterials and flexible sensors, which enables real-time detection of various full-body movements using the combination of a wireless sensor suit and deep-learning-based cloud computing. This system includes an array of a nanomembrane, laser-induced graphene strain sensors, and flexible electronics integrated with textiles for wireless detection of different body motions and workouts. With multiple human subjects, we demonstrate the system’s performance in real-time prediction of eight different activities, including resting, walking, running, squatting, walking upstairs, walking downstairs, push-ups, and jump roping, with an accuracy of 95.3%. The class of technologies, integrated as full body-worn textile electronics and interactive pairing with smartwatches and portable devices, can be used in real-world applications such as ambulatory health monitoring via conjunction with smartwatches and feedback-enabled customized rehabilitation workouts.

## 1 Introduction

The recent progress in materials, manufacturing technologies, and the integration of artificial intelligence contribute to promoting wearable bioelectronics in addressing human healthcare applications. In particular, real-time prediction of human intention for movements can offer new opportunities for the healthcare and rehabilitation industry since it provides valuable insights into the users’ everyday activities in real time. The latest advances in wearable strain sensors have demonstrated significant progress in the precise detection of human movements with high sensitivity,^1−5^ but limitations in materials design and integration hinder their realistic applicability for human healthcare technologies. Primarily, the prior strain sensors do not incorporate the portable circuitry for the continuous wireless collection of strain data, which makes them unable to detect human motion in real time.^1,2,5−11^ Besides continuous motion detection, a majority of the existing studies cannot predict intended human movements due to the absence of a machine learning model to classify multiple classes of motions in real time.^12−19^ Furthermore, several studies classified the multiple classes of human gestures based on strain information on the specific body parts such as hand^4,20,21^ and throat.^22,23^ Indeed, decoding human gestures from local body parts yields valuable information, but expanding the scope to encompass the entire body could provide novel insights into human healthcare monitoring. Predicting the intended full-body motion opens the possibilities for continuous examination of the users’ bodily activities as it can transform real-life applications such as human healthcare,^24^ rehabilitation,^25,26^ sports biomechanics,^27^ and human-machine interactions.^28^ To address these challenges, recent advancements in materials engineering, including flexible electronics and textile-integrated designs, provide a foundation for wearable systems capable of full-body motion recognition. Nevertheless, the existing textile-integrated strain sensors fail to provide portable wireless detection of full-body motion and recognition of real-time human motions via machine learning.^29−31^ Overall, the prior studies lack the integration of all of the functional components that are essential for realizing the practical applicability of the wearable sensor suit in real-life scenarios.

Here, we introduce fully body-worn textile-integrated electronics for wireless, real-time motion recognition using cloud-based machine learning. The electronic system comprises multiplexed nanomembrane graphene sensors and a flexible circuit such that the bodily strain information can be collected and then wirelessly transmitted to the cloud for continuous motion recognition. Besides, the Velcro-mesh textile is incorporated into soft electronics such that it can be ergonomically attached to the motion-predicting suit, allowing easy adjustment even for human subjects of different body sizes. In addition, cloud-based deep learning processes multiplexed body strain data to predict the type of workout that the user intends to perform in real time. This wearable sensor suit can recognize eight classes of intended workouts with real-time classification. Thus, to further enhance the real-world applicability of the sensor suit, we pair the wearable sensor suit with a smartwatch and other portable devices via Bluetooth so that it can generate personalized workout feedback along with calorie calculation. In this context, we expect that the textile sensory electronics delivered by this study will transform extensive healthcare applications and rehabilitation science since such a new type of full-body textile electronics with realistic functionalities has not been reported.

## 2 Result and Discussion

### 2.1 Overview of a Full-Body, Textile-Integrated, Soft Electronic System for Wireless, Real-Time, Continuous Motion Recognition Using Cloud Computing

**Figure 1:**
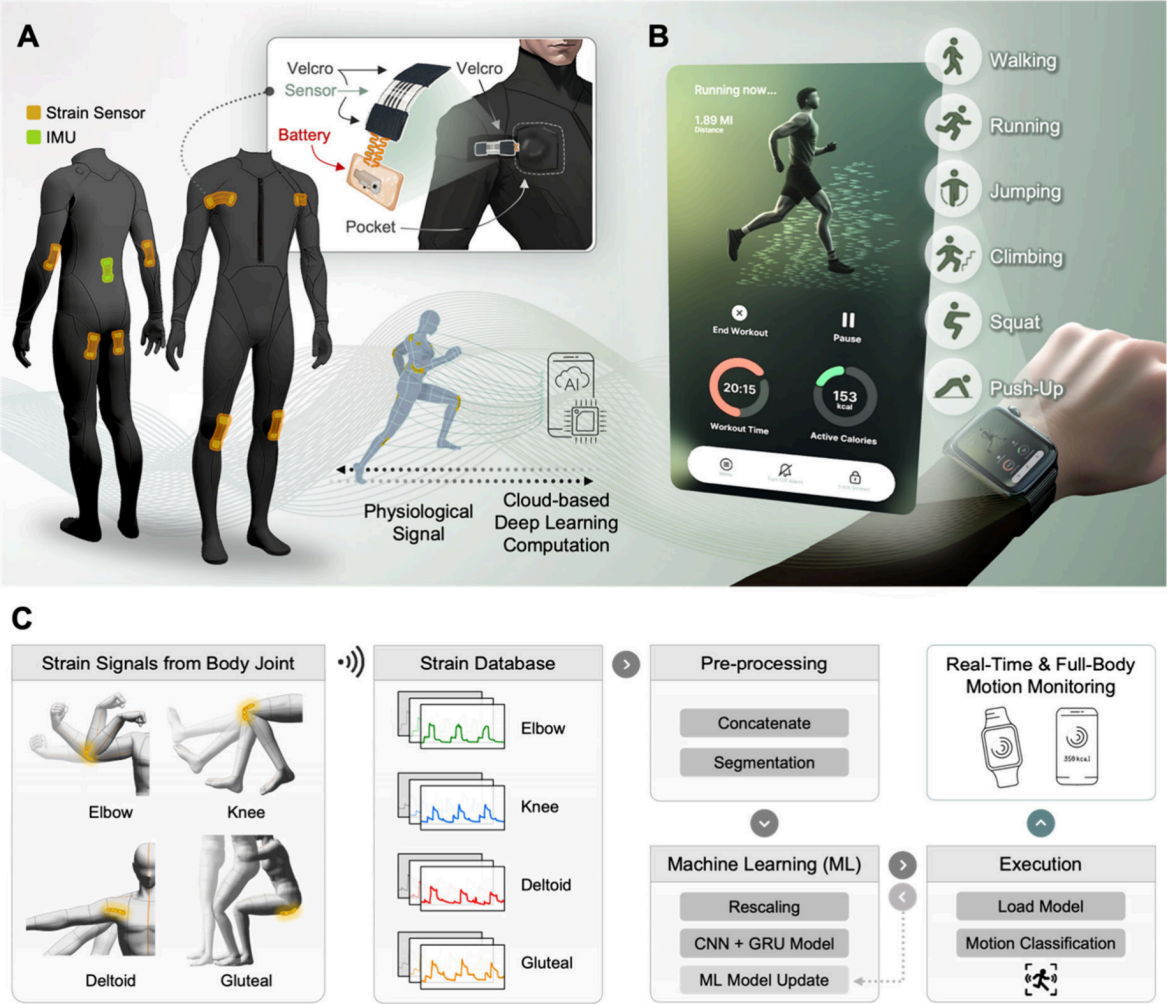
**Overview of full body-worn textile-integrated soft microelectronics for real-time continuous motion recognition using cloud computing**. A. Graphical representation of the multiplexed strain sensing suit. B. Virtual example of the graphic user interface of the smartwatch when coupled with the strain sensing suit. C. Flowchart that exhibits the general motion predicting flow from body movement, strain sensing, preprocessing, and machine learning to the graphic user interface of the portable device.

Figure 1A delineates the graphical representation of the multiplexed laser-induced graphene (LIG) strain suit, along with the textile-integrated strain sensor unit in the inset. The strain suit is comprised of eight wireless soft strain sensors and an inertial measurement unit (IMU) sensor that are attached to the deltoids, elbow, gluteal, knee, and back to acquire dynamic information about the human body. We incorporated the IMU sensor (at the back) that sets a reference point in the three-dimensional space, allowing continuous monitoring of the user’s spatial orientation. The wireless strain sensor mainly consists of a flexible circuit, a LIG sensor unit, Velcro patches, and interconnecting electrodes. The flexible polyimide (PI) circuit collects the real-time body strain data and wirelessly transmits the data to the cloud system, where the deep learning model is uploaded to classify the type of workouts. The serpentine interconnection electrode, which is made up of three layers of PI, Cu, and PI, connects the wireless circuit and sensor unit. Velcro patches positioned at both extremities of the sensor unit facilitate effortless attachment to the suit, rendering it adaptable and functional for users of diverse body sizes. Thus, upon integration with the smartwatch, as illustrated in Figure 1B, the device obviates the need for manual selection of the workout type by the user, because the deep learning model will automatically recognize the ongoing workout. Thus, users can seamlessly engage in their workout routines with the smartwatch autonomously calculating the corresponding calorie expenditure. Figure 1C illustrates the flowchart that elucidates the methodology for processing strain data from individual body parts to predict the intended user movements. Specifically, during the execution of a specific workout by a user in the suit, diverse combinations of strain signals emerge across six body joints. The multiplexed strain database information is subsequently uploaded wirelessly to the cloud-based platform, where it undergoes processing through machine learning algorithms. The processing stage initiates with preprocessing, which is composed of segmentation and rectification of strain signals, which are transformed into inputs suitable for machine learning. Then, the machine learning protocol rescales the strain signals from eight channels with the time stamp, so that the signal data can be used to train the machine learning model. The significance of cloud-based machine learning arises from accessibility to the extensive quantity of the strain database, facilitating iterative training of the machine learning model and, thus, making the prediction more accurate. Moreover, the cloud platform allows developers to update the machine learning model from any location at any time because accessibility to the cloud is unrestricted. Finally, through integration with the smartwatch, users are informed about the intended workout during the exercise session without the need for manual selection via the device interface.

### 2.2 Characterization of LIG Electrode for Strain Sensors

**Figure 2:**
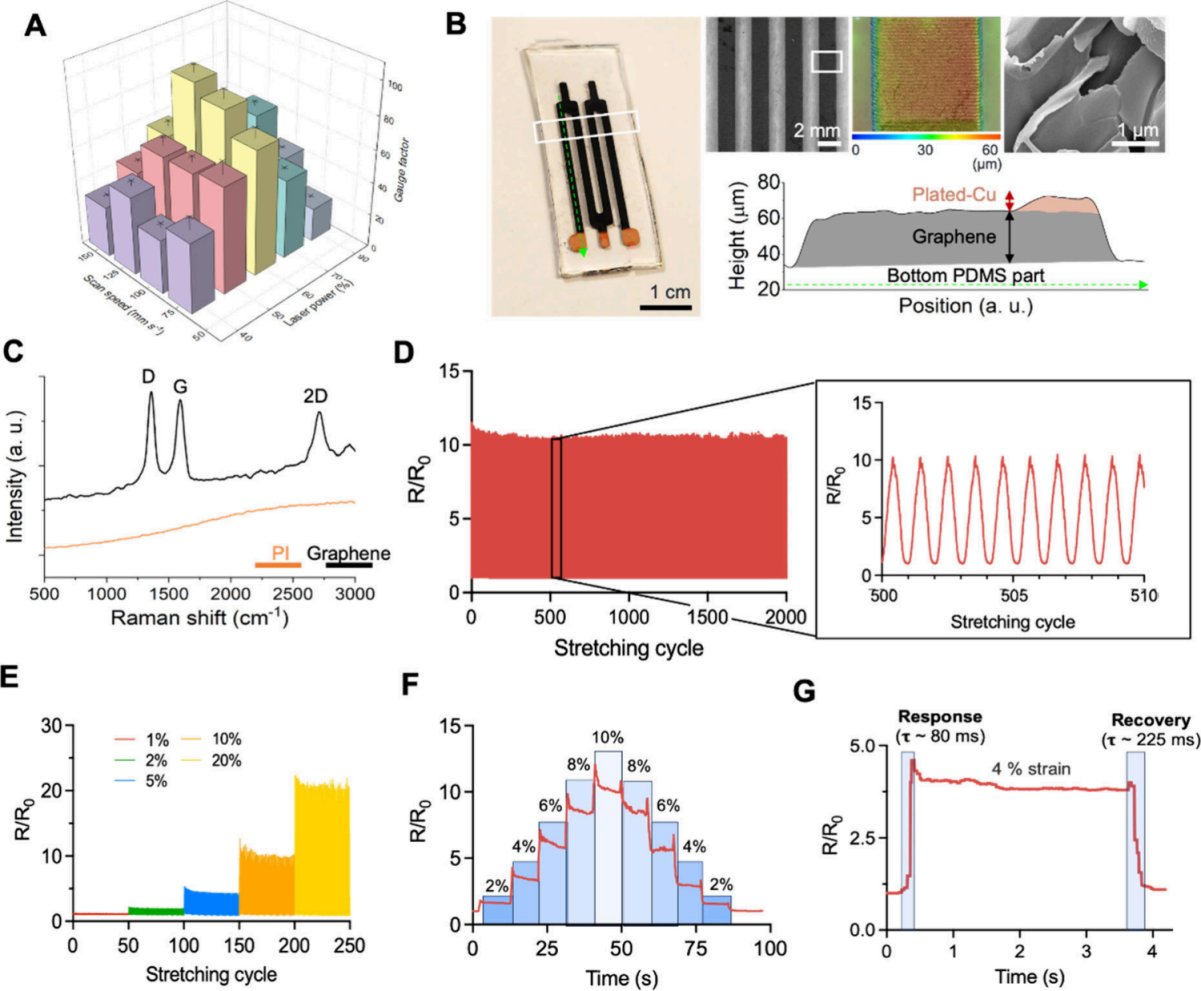
**Characterization of LIG electrode for strain sensing**. A. Laser parametric study to derive laser irradiation condition with the largest gauge factor. B. Actual photograph of the LIG electrode with the Cu-plated contact pad. The top three images correspond to an optical microscopy image, a surface profile image, and a scanning electron microscope image of the LIG electrode in use. The image in the bottom-right corner shows the height of the LIG sensor with the Cu-plated pad in relation to the length position. C. Raman spectra of the LIG electrode. D. Normalized resistance change of the repeated 2000 strain cycles of 10% strain. The inset figure magnifies the strain cycles in the middle of the cyclic test. E. Stepwise normalized resistance change of the LIG sensor with the 50 strain cycles of 1%, 2%, 5%, 10%, to 20%. F. Stepwise normalized resistance change of the LIG sensor with the increasing and decreasing trend at 2%, 4%, 6%, 8%, and 10%. G. Response and recovery of the LIG sensor under 4% strain.

To determine the optimal laser parameters to achieve a high gauge factor (GF), we conducted a parametric study in which we varied the laser scanning speed and laser intensity since these parameters mainly contribute to the laser energy density (Figure 2A). The GF was measured using a standardized procedure. The LIG strain sensor was mounted on a motorized stretching platform, where incremental strains (e.g., 5%, 10%, 20%) were applied in a controlled environment. The resistance changes were continuously recorded using an LCR meter during stretching and releasing cycles. The GF was calculated using the following formula:
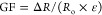
where Δ*R* is the change in resistance during strain, *R*_0_ is the initial resistance of the sensor, and ε is an applied strain

The measurement was repeated for multiple sensors fabricated under identical conditions to ensure repeatability and reliability. We measured the GF of all of the parametric study samples, and the result suggests that the sample condition with 125 mm s^–1^ and 7.5 W generates the largest GF of 96.35 ± 4.62. Human body motions typically involve strain levels ranging from 0% to 20%, such as joint flexion, muscle contractions, and postural adjustments. A high GF enables precise detection of these strain variations, allowing the sensor to differentiate between subtle and large movements effectively. This sensitivity is essential for resolving distinct temporal and spatial strain patterns across various activities, such as walking, running, and squatting, achieving sufficient GF to cover the human body strain.^5^ The laser scanning speed and intensity are crucial parameters for determining the energy density delivered during the laser-induced graphene LIG fabrication process. These parameters directly influence the structural and morphological properties of the graphene layer, which in turn affect the GF values of the resulting strain sensor. The high laser energy density condition usually generates high GF values as it produces the extremely porous nanomicrostructure of LIG that contributes to (1) a reversible contact change in the graphene network under strain, and (2) varied hopping/tunneling effects,^32,33^ However, excessive laser energy density during the LIG fabrication can damage the PI substrate by ablation. Moreover, overly high energy disrupts the hierarchy, leading to compacted structures that limit tunneling effects and reversible contact changes that are essential for achieving a high GF. In this regard, an optimal laser-processing condition exists to produce a graphene electrode with a high GF, which yielded 96.35 ± 4.62.

Figure 2B shows the actual snapshot of the optimized LIG sensor electrode that has been transferred from the PI film to the polydimethylsiloxane (PDMS) layer. The sensor electrode design adheres to a conventional uniaxial strain gauge configuration with a total length of 25 mm, as exhibited in the inset figure. The optical and scanning electron microscope images in the insets deliver multiscale views of LIG, which can be characterized by the hierarchical porous microstructure that enhances sensitivity, as discussed earlier. The surface profile in color indicates the approximate height of the LIG electrode which corresponds to 59.6 ± 5.5 μm (Figure S1). The shaded area in pink represents the electroplated Cu on the surface of the LIG electrode to facilitate conventional soldering with the interconnects. Figure S2 also presents the top and cross-sectional views of the transferred LIG electrode on the PDMS substrate in which the unshaded region corresponds to the LIG electrode. The EDX mapping in Figure S2B also confirms the chemical composition of the electrode, PDMS, and e-plated Cu. In Figure 2C, Raman spectroscopy provides crucial insights for discerning the presence of graphene after laser irradiation on the PI substrate. The Raman spectrum of graphene exhibits distinctive peaks, including the D band (approximately 1340 cm^–1^), G band (approximately 1585 cm^–1^), and 2D band (approximately 2696 cm^–1^), as indicated in the figure. Typically, when assessing graphene through Raman spectroscopy, the D band spectrum indicates the presence of either sp^2^ hybridization C bonds or defects, while the G band corresponds to first-order phonons, and the 2D band signifies second-order boundary phonons.^34^ The I_2D_/I_G_ ratio of 0.842 suggests the formation of single-layer graphene since the ratio is considerably lower than 2. Figure S3 provides Raman spectra curves for all of the parametric sample conditions of different laser power and scanning speed. The result corroborates the negligible shift of the D bands and the existence of single-layer graphene for all parametric LIG conditions.^35^ To examine the mechanical durability and strain sensing reproducibility, we applied a cyclic strain of 10% to the sensor electrode for 2000 repeated cycles, as in Figure 2D, which also exhibits the magnified stretching cycles in the inset. The consistent normalized resistance throughout all the cycles substantiates the mechanical robustness and data reliability after repeated use. Figure 2E illustrates the stepwise normalized resistance change in the LIG sensor across 50 strain cycles, with strain levels progressively increasing from 1%, 2%, 5%, 10%, to 20%. Furthermore, Figure 2F details how the resistance of the LIG sensor varies with a stepwise increase and then decreases in strain levels, specifically at intervals of 2%, moving through 4%, 6%, and 8%, and culminating at 10%. These observations indicate a direct proportional relationship between the normalized resistance and the magnitude of strain, highlighting the sensor’s ability to reliably differentiate between varying magnitudes of strain through changes in resistance. Besides, when the sensor was stretched by 10% of strain and returned to the original state, we were able to measure the fast response and recovery time of 80 and 225 ms, respectively (Figure 2G). The immediate response and recovery rate of the strain sensor are essential particularly in this work because the sensors need to monitor the real-time strain information on the human subject, and the time delay in response or recovery might exert a critical effect on the continuous machine learning classification accuracy.

### 2.3 Fabrication of Soft Integrated Electronics Using LIG Strain Sensors and Flexible Circuits

**Figure 3:**
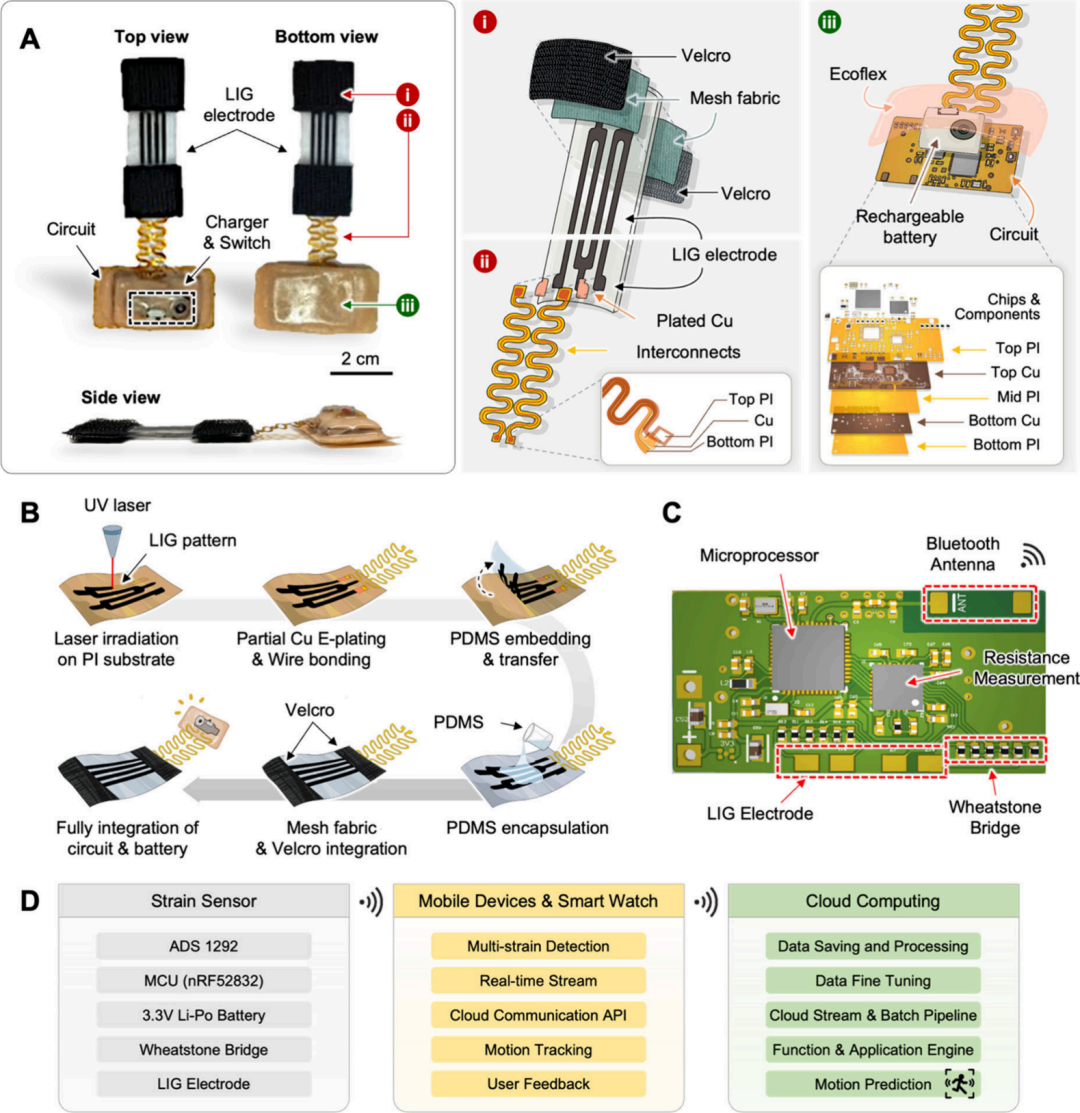
**Integration of LIG strain sensor with functional components**. A. Top, bottom, and side views of the complete LIG strain sensor with the integrated components of LIG electrode, Velcro-mesh fabric, interconnects, and flexible circuit. The inset figures show the exploded view of the sensory unit (left) and circuit unit (right). B. Graphical representation of the fabrication process of the integrated strain sensor. C. Top view of the flexible circuit with the labeled integrated circuit (IC) components. D. Flowchart, including a strain sensor, portable devices, and cloud computing.

**Figure 4:**
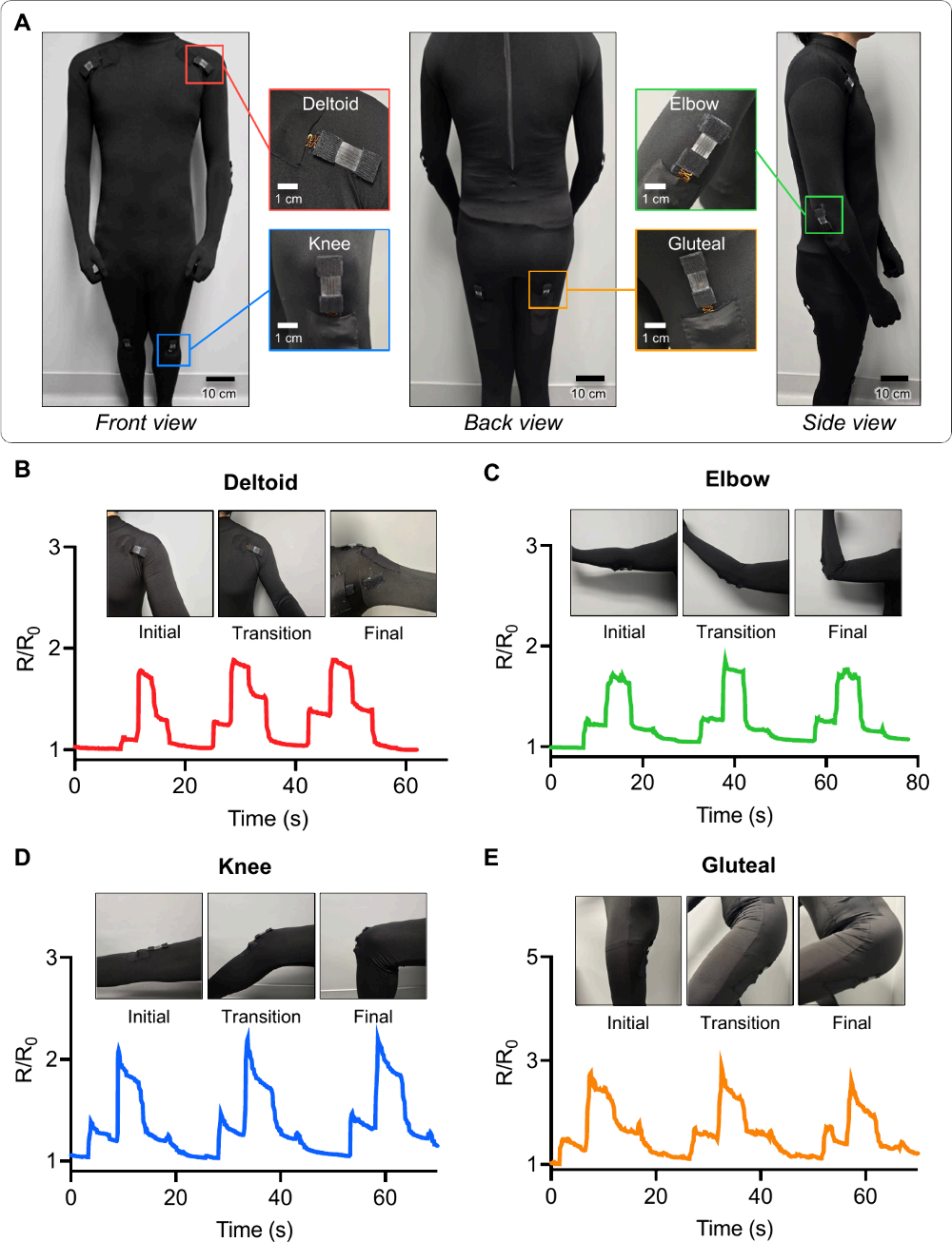
**Multiplexed strain sensing suit with joint movement monitoring.** A. Front, back, and side views of the human subject wearing the strain sensing suit with the magnified view of strain sensors on the deltoids, knee, elbow, and gluteal. B-E. Joint movement monitoring with the normalized resistance change of deltoid (B), elbow (C), knee (D), and gluteal (E).

Figure 3A shows the integrated design of the soft LIG strain sensor unit, which is mainly composed of the LIG electrode, Velcro-mesh fabric, interconnects, and flexible circuit. The Velcro-mesh fabric facilitates facile attachment to the suit, while the textile-integrated design enhances ease of location adjustment since it can be easily detached and adjusted. Moreover, the ergonomic sensor design, which is capable of physical separation from the suit, comprehensively accommodates individuals with diverse body sizes and shapes, making the system fit for all. The serpentine interconnects establish electrical connections between the LIG electrode and the flexible circuit, mitigating potential noise from motion artifacts. The flexible circuit incorporates the customized, onboard battery that can be recharged with the magnetic cord, thereby achieving a fully untethered design, and eliminating noise associated with tension forces from wires for the power supply. Figure 3B illustrates the individual procedure steps to fabricate and integrate the LIG strain sensor unit. First, the pyrolytic ultraviolet (UV) laser irradiation on the PI substrate converts PI into graphene, and we electroplated copper on the contact pad of the LIG electrode for electrical connection with the serpentine interconnects. Then, PDMS embedded on the LIG electrode allows facile transfer of the electrode to the PDMS substrate, and we encapsulated the other side of the LIG surface with a thin film of PDMS as a protective layer. Lastly, the incorporation of the Velcro-mesh fabric and flexible circuit into the LIG electrode completes the fabrication process of the LIG strain sensor. The flexible circuit of the strain sensor as in Figure 3C includes the essential components for continuous data recording and wireless data transfer. The integrated circuit (IC) chip for resistance measurement (ADS 1292) and an array of Wheatstone bridges enable a precise measurement of the low resistance while the microprocessor unit (MPU) and Bluetooth antenna wirelessly transfer the real-time recorded resistance data acquired from the LIG electrode. Furthermore, incorporated into the flexible circuit for IMU monitoring as in Figure S4, the commercial IMU sensor (ICM 20948) provides the user’s spatial orientation in the three-dimensional space, and the MPU enables wireless data transfer and real-time data acquisition just as the strain sensor unit. Figure 3D depicts the seamless integration of the LIG strain sensor with digital technologies for comprehensive motion tracking and enhanced user feedback. The system employs a mobile application, Google’s Flutter framework, which ensures broad compatibility with both iOS and Android operating systems. This application is pivotal for real-time data collection, local storage, and cloud synchronization of data from a wearable device. Cloud computing infrastructure supports such an ecosystem, as it incorporates both stream and batch data processing capabilities along with an application engine utilizing FastAPI for sophisticated motion prediction algorithms. The architecture herein enables efficient data management and processing, optimizing the system for predictive analytics. The synergy between wearable technology and cloud computing facilitates real-time monitoring and advanced health analytics, positioning this system as a cutting-edge solution for remote health monitoring applications. Figure 4A demonstrates a human subject in a multiplexed strain-sensing suit with multichannel strain sensors attached to the deltoids, knees, elbows, and gluteal to obtain the strain information on the body joints. As the human subject performs shoulder abduction (Figure 4B), elbow flexion (Figure 4C), knee flexion (Figure 4D), and squatting (Figure 4E) to generate the mechanical strain at the corresponding joints, the normalized resistance of the sensor of each location quickly changes. The sensors on the elbow and deltoid reliably measure motion ranges of up to 145° and 180°, respectively. Sensors on the knee and gluteal measure range up to 140° and 125°, respectively. This capability allows comprehensive full-body motion tracking, accommodating large and subtle strain changes. The real-time resistance response of the strain sensor during the movement suggests that the incorporation of machine learning would allow us to recognize the user’s intended workout based on the multichannel strain pattern.

### 2.4 Demonstration of a Full-Body, Textile-Integrated Electronic System for Real-Time, Continuous Motion Recognition Using Cloud Computing

**Figure 5:**
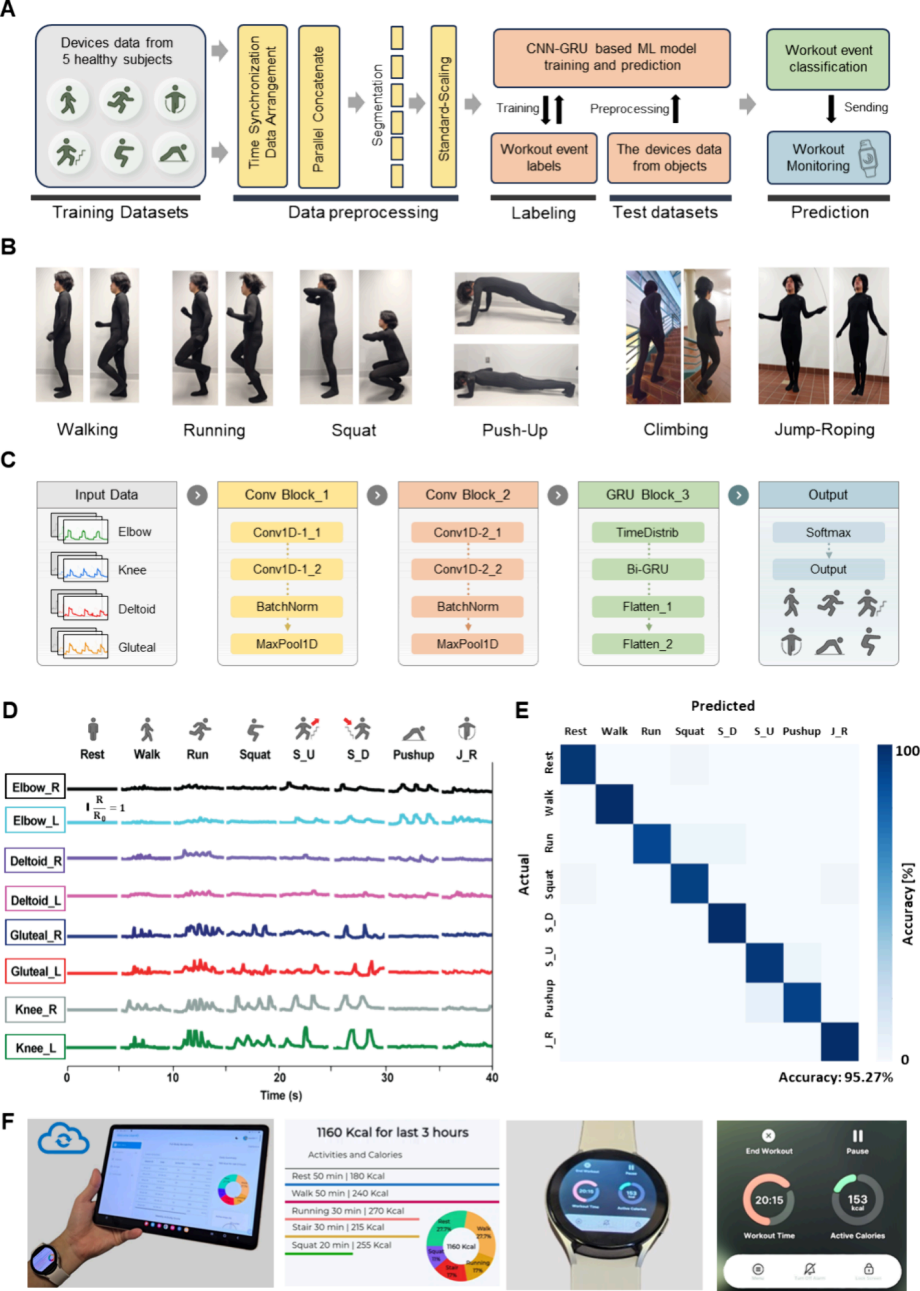
**Demonstration of a full-body textile-integrated electronic system for real-time, continuous motion recognition using cloud computing**. A. Flowchart of the overall process for motion recognition from training data sets, data preprocessing, machine learning, and prediction of outcomes. B. Photos of a subject who wears a full-body suit with electronics, making different motions via walking, running, squatting, push-ups, climbing, and jump roping. C. Flowchart of deep-learning architecture for predicting the intended movement of the user in real-time. D. Measured electrical resistance changes from sensors mounted on different body parts according to the subject’s different activities, including resting, walking, running, squatting, walking upstairs, walking downstairs, push-ups, and jump roping. E. Confusion matrix of predicting the intended movement of the user, showing 95.3% accuracy in classifying 8 classes in D. F. Interactive pairing with a tablet and a smartwatch that display a personalized workout report.

In this study, we have developed a daily activity monitoring method through a sophisticated hybrid deep learning model that combines a convolutional neural network (CNN)-gated recurrent unit (GRU) architecture. This approach merges the spatial feature extraction discernment of CNNs with the sequential data processing capabilities of GRUs (a variant of long short-term memory or LSTM), providing improved precision and dynamic evaluation in real-time scenarios.^36,37^Figure 5A epitomizes the graphic flowchart of real-time motion recognition from data acquisition to the final prediction phase. We collected full-body motion data from five healthy participants to train the model. While wearing a multiplexed strain suit, each participant conducted seven distinct exercises, as shown in Figure 5B, which captured **the initial and concluding postures for each exercise, visually presenting** the target movements. The differentiation between movements such as the squat and lunge is achieved through a temporal sequence and spatial correlation analysis across multiple sensors. For example, although both movements involve knee bending, the temporal activation sequence and compensatory motions in other body parts differ. Furthermore, the inclusion of the IMU sensor provides spatial orientation data that contextualize the strain signals, offering an additional layer of differentiation. After initial preprocessing, the deep learning model executes epoch-by-epoch data consisting of 5 s long filtered strain and IMU data. Figure 5C presents the CNN-GRU hybrid model architecture that processes data from wearable strain sensor patches for a full-body motion analysis. As a preliminary step, data normalization ensures that all strain and IMU signals are scaled between 0 and 1. The data set is then partitioned into training, validation, and testing segments following a 60:20:20 ratio. Our model employs parametric rectified linear units (PReLU) for activation, which can optimize the network with the ADAM optimizer and calculate losses via the categorical cross-entropy function. Hyperparameters were fine-tuned through a random selection methodology with an early stopping functionality that is implemented upon detecting no improvement over two consecutive evaluations. The architecture of our model features two 1D convolutional layers, each enhanced with Batch Normalization (BN) and PReLU activation, followed by a GRU layer and two dense layers with dropout to mitigate overfitting. The final layer, a softmax activation, classifies the data into eight categories (rest, walk, run, squat, stair up, stair down, push up, jump-rope) based on distinct signal features, as illustrated in Figure 5D (strain signal) and Figure S5 (IMU signal). Further details on the model’s layers and parameters are available in the Experimental Section. The resulting confusion matrix, as depicted in Figure 5E, shows a high accuracy rate of 95.27%. Movie S1 illustrates a real-time movement prediction demonstration, continuously transitioning from a state of rest to each of the trained workouts at every ten-second interval. Figure S6 exhibits the screenshot images of the cloud-based GUI, offering visual insight into the interface that is used for real-time movement analysis and feedback. Textile electronics can be paired with smartwatches and other portable devices to predict the user’s intended workout and calculate the calories burned in real time. Smartwatches can inform the user about the number of calories burned when the user carries out a specific type of workout based on IMU information.^38,39^ However, these smartwatches cannot accurately distinguish the specific type of ongoing workout based on the single-channel IMU information. On the other hand, the wearable sensor interface also automatically generates reports for calorie calculations and activity tracking, based on the real-time classification from our model (Figure 5F and Movie S2). The system calculates energy expenditure using
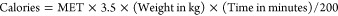
where MET values are calibrated for each activity (Resting: 2.9, Walking: 3.9, Running: 7.4, Stair climbing: 5.9, Squatting: 10.4). For a 70 kg individual, this translates to realistic energy expenditures, such as walking (240 kcal/50 min) and running (270 kcal/30 min). The calibrated MET values are automatically applied based on the real-time movement classification, enabling accurate calorie tracking throughout the workout session. Thus, the wearable sensor suit enables activation-based personalized feedback and facilitates long-term workout outcomes. The demonstration in this work highlights the system’s capacity to serve as an effective instrument for the real-time observation of daily activities, making notable advancements in the domains of health surveillance and rehabilitation science. As shown in Table 1, our system’s capabilities surpass existing technologies in wireless connectivity, multimotion recognition, and cloud integration, establishing it as a unique solution for real-time health monitoring and personalized rehabilitation.

**Table 1:** Performance Comparison between the Full Body-Worn Electronics and Prior Work Detecting Human Motions

	All-in-one wireless strain sensor for full body motion detection	Cloud computing	Interactive pairing with smartwatches/portable devices	Capable of predicting user’s intended movements	Direct textile integration	# of sensors	# of motion
**This work**	**O**	**O**	**O**	**O**	**O**	**9**	**8**
(40)	X	X	X	X	X	5	6
(41)	X	X	X	X	X	1	2
(42)	X	X	X	X	X	1	2
(43)	X	X	X	X	X	5	10
(44)	X	X	X	X	X	1	2
(45)	X	X	X	X	X	1	2
(5)	X	X	X	X	X	18	3
(46)	X	X	X	X	X	1	4
(47)	X	X	X	X	O	1	2
(48)	X	X	X	X	O	1	3
(49)	X	X	X	X	O	2	4
(21)	X	X	X	O	X	2	9
(50)	X	X	X	O	X	3	7
(51)	X	X	X	O	X	1	13
(4)	X	X	X	O	O	4	6
(52)	X	X	X	O	X	2	6
(53)	X	X	X	X	X	46	10
(54)	X	X	X	X	X	5	5

## 3 Conclusion

This paper introduces a multiplexed, soft, wearable strain-sensing suit that can recognize the user’s real-time workouts by strain information based on machine learning on the cloud platform. The fabricated LIG electrodes in full body-worn textiles show a high gauge factor and rapid response/recovery for continuous real-time motion-predicting application. Unlike the conventional strain sensors, this work’s textile electronics integrate a wireless flexible circuit and an array of membrane strain sensors with a soft mesh patch. These materials-centric advancements allow user comfort, reduced motion artifacts, and complex motion recognition via wireless signal detection. Cloud-based machine learning, recognizing the user’s various workouts, shows an accuracy of 95.27% with eight classes. With smartwatch integration, the wearable sensor suit enables the automatic selection of workout types. At the end of each workout, the system provides comprehensive reports detailing activity outcomes and energy spent. This study, highlighting advancements in materials engineering, system integration, and cloud computing, will offer significant insights into developing various soft intelligent electronics for advancing smart exercises and smart healthcare.

## 4 Experimental Section

### 4.1 LIG Electrode Fabrication

A PI tape was adhered to a glass slide, followed by the generation of LIG using a UV laser (Alabama Laser) as shown in Figure S7. An electroplating was performed by supplying a current of 0.06A for 5 min using a power supplier (Keithley 2200 DC Power Supply). Figure S8 explains the method used to electroplate Cu to facilitate the soldering with the interconnects. A copper pad was created at one end of the LIG electrode, to which an interconnector was soldered for connection. PDMS (Sylgard 184, Dow) was poured and spin-coated at 100 rpm and cured at 100 °C for 20 min. After transferring the LIG electrode onto PDMS, the second layer of PDMS was spin-coated at 50 rpm and cured at 100 °C for 20 min to encapsulate the electrode.

### 4.2 Fabric Substrate Fabrication

Silbione (A-4717, Factor II Inc.) parts A and B were mixed in a 1:1 weight ratio for 10 min. This mixture was spin-coated onto a polytetrafluoroethylene (PTFE) sheet at 1200 rpm for one min to ensure a uniform adhesion layer thickness. The surface was then covered with brown fabric medical tape (9907T, 3M) and cured in an oven at 65 °C for 30 min. After curing, the PTFE sheet was detached.

### 4.3 Circuits and Interconnector Fabrication

A flexible PCB (fPCB) was employed on which all electronic components were mounted using a reflow solder process. Laser cutting was utilized to remove unnecessary areas, enhancing the mechanical flexibility of the circuit. A lithium polymer battery assembly with a slide switch and a circular magnetic recharging port was incorporated for the power supply and management. A low-modulus elastomer (Ecoflex Gel, Smooth-On) was placed underneath the integrated circuit to isolate the strain. The entire electronic system was encapsulated with an additional elastomer (Ecoflex 00–30, Smooth-On), leaving only the switch and charging port exposed.

### 4.4 Soft Sensor Assembly

The interconnector was soldered to the circuit. The soft-packaged electronic system (circuit part) was then attached to the fabric side of the fabric substrate by adding and curing a thin layer of silicone, completing the assembly process. The electrode was combined with Velcro (VELCRO Brand) using silicone adhesive (Gorilla) and fabric meshing. Figure S9 demonstrates the fabrication process for integration of the strain sensor that consists of the LIG electrode, circuit, interconnects, and Velcro-mesh patches.

### 4.5 Soft Sensor Characterization

The testing setup for both mechanical and electrical assessment included utilizing a digital force gauge (M5–5, Mark-10) in conjunction with a motorized testing platform (ESM303, Mark-10) for evaluating mechanical properties, as well as an LCR meter (Model 891, BK Precision) to quantify electrical resistance (Figure S10). To evaluate cyclic stretching, the electrode system underwent alternating stretching and releases in a vertical direction at a rate of 10 mm/min, repeated for 2000 cycles. In contrast, this stretching and releasing were conducted for 50 cycles at the same rate as that of the stepwise normalized resistance assessment. The gauge factor (GF) value was calculated by using the following equations: GF = , where Δ*R* is the change in resistance, *R* is the nominal resistance of the strain gauge, and ε is the strain. For a detailed investigation of the microstructural characteristics and elemental composition of the as-prepared LIG electrodes, a Field Emission Scanning Electron Microscope equipped with Energy Dispersive X-ray Spectroscopy (FE-SEM/EDS, SU8230, Hitachi) was utilized. Raman Spectroscopy (Qontor Dispersive Confocal Raman Spectrometer at a wavelength of 488 nm, Renishaw) was employed to identify the presence of prominent peaks (i.e., D, G, and 2D) within the LIG patterns. A simple observation was conducted by means of a digital microscope (VHX-7000, Keyence).

### 4.6 Data Processing and Acquisition with Firmware

Data processing within our full-body motion tracking system is initiated at a low-power microcontroller (nRF52832, Nordic Semiconductor), which is programmed with a Bluetooth system-on-a-chip for efficient data transmission. This microcontroller interfaces with the ADS1292, a low-power analogue front-end (AFE) sensor, through the Serial Peripheral Interface (SPI). A Wheatstone bridge circuit is also utilized, allowing the microcontroller to accurately measure the electrical resistance values. The system employs an 18-bit Analog-to-Digital Converter (ADC) to transmit digital signals, where the ADC’s output decimal value corresponds to the measured resistance value and range. The nRF52832 incorporates a SoftDevice, which is a precompiled and linked binary firmware optimized for wireless communication protocols. This setup enables the microcontroller to wirelessly transmit data to a custom app-installed mobile device (Galaxy Tab S8 tablet, iPad Pro fourth generation, Apple Watch 7, and Galaxy Watch 5) via Bluetooth Low Energy (BLE), ensuring efficient and seamless communication. The strain sensor circuit is powered by a 110 mAh rechargeable lithium-polymer battery, supporting extended operation periods of 11 h or more. Furthermore, 8 wearable sensors are identified by distinct MAC addresses of the microcontroller unit (MCU) and peripherals (Figure S11). This design allows the mobile system to capture and process data from multiple devices in real-time seamlessly, and is capable of providing a comprehensive motion monitoring system.

### 4.7 Data Cloud Computing Interface and Mobile System

The system initiates with the connection of 8 devices, each designed with strain sensors adept at capturing comprehensive body movements. These multiple 10hz sampling strain sensor data are compiled into bulk data files and wirelessly transmit data to an authentication and computation server at 5-s intervals. The data uploading is efficiently managed by FastAPI, which serves as a bridge between the hardware and Google Cloud Platform (GCP). The data are preprocessed and converted to a (50,8) matrix of segmented waveform information. Standardization and normalization are applied to input data for the variance detection of the learning model. Within GCP, a deep learning model classifies data against predefined physical actions or exercises using a computation engine designed for rapid processing. For real-time systems with request and post speeds of under 200 ms, the system provides feedback and motion classification. For effective feedback with the mobile system, we employed Flutter Dart due to its capability to compile into native ARM or Intel x64 code for creating an efficient cross-platform application that operates seamlessly on both Android and iOS infrastructures. Unlike React Native and Xamarin, which often rely on platform-specific UI components, Flutter enables the UI/UX development of visually consistent applications across platforms without the need for such dependencies. Both ahead-of-time (AOT) and just-in-time (JIT) framework supports processes in real-time and asynchronous processes in excellence. The application is configured to support multiple sensor data processing and asynchronous handling simultaneously. Remotely customizable analysis was programmed for both immediate analytical insights and the compilation of daily and monthly statistics for thorough long-term monitoring.

### 4.8 Classification of Full-Body Movement

In our research on full-body movement patterns using strain and IMU signals, we employed a deep learning architecture that integrates both CNN and GRU layers. This model was constructed using TensorFlow 2.0 in Python and executed on a laptop equipped with an Intel i7 processor (I7–9750H). The IMU and strain signals (10 Hz), segmented into 5 s intervals, underwent a normalization process to have values ranging between 0 and 1 using the Standard Scaler method. We strategically divided our data set: 60% was allocated for training, 20% for validation, and the remaining 20% for testing. The architecture begins with an input layer shaped to the signals, followed by two convolutional layers: the first with 64 filters (kernel size 3) and the second with 32 filters (kernel size 5), each accompanied by an additional convolutional layer with 4 filters (kernel size 4), PReLU activation, and max pooling. These layers feed into a time-distributed layer with 64 units and a bidirectional GRU layer with 40 units, recurrent dropout (0.1), and tanh activation. Subsequent layers include a dense layer of 96 units with PReLU activation, another dense layer with 160 units and PReLU activation, a 0.2 dropout layer, and an output dense layer using softmax activation. For the optimization process, we utilized the ADAM optimizer with a fixed learning rate of 0.001, and losses were computed by using the categorical cross-entropy function. Throughout the training, the weights of the model were dynamically adjusted based on validation accuracy, and an array of hyperparameters was selected through a Keras Tuner’s random search method. The ultimate model, showing the highest validation accuracy, was then put to the test to gauge its predictive ability (Figure S12 and Table S1).

## Data Availability

The data that support the findings of this study are available from the corresponding author upon reasonable request.

## Supporting Information Available

Figure S1. Height profile of LIG electrode; Figure S2. Microstructure of LIG electrode; Figure S3. Raman spectra of laser-induced graphene (LIG); Figure S4. Wearable IMU sensor; Figure S5. IMU pattern of all joint movements for each workout; Figure S6. Screenshots of the cloud-based Android GUI; Figure S7. Laser printing setup for the LIG electrodes; Figure S8. Electroplating setup for the interconnector of LIG electrodes; Figure S9. Wearable strain sensor fabrication process; Figure S10. Mechanical test setup for the LIG strain sensor electrodes; Figure S11. Schematic illustration of the device on power, hardware structure, data, and the real-time motion monitoring system; Figure S12. Machine learning architecture for full-body motion classification; Table S1. Machine learning layer information for full-body movement classification (PDF)
Movie S1. Real-time full body motion recognition (MP4)
Movie S2. Key features of the Strain sensor electronics system (MP4)

## Notes

**Human subject study**. The study involved multiple volunteers aged 18 or older, and the study was conducted by following the approved Institutional Review Board protocol (Protocol-H24090) at Georgia Institute of Technology. Before the in vivo study, all subjects agreed to the study procedures and provided signed consent forms.
