# Supplementary material for: Full Body-Worn Textile-Integrated Nanomaterials and Soft Electronics for Real-Time Continuous Motion Recognition Using Cloud Computing
Source: ACS Appl Mater Interfaces. 2025 Jan 24;17(5):7977–88. doi: 10.1021/acsami.4c17369 (PMC11803620; doi:10.1021/acsami.4c17369)
Supplement: Supplementary file 1 — am4c17369_si_001.pdf [file am4c17369_si_001.pdf]

Supporting Information

Full body-worn textile-integrated nanomaterials and soft electronics for real-time continuous motion recognition using cloud computing

Kangkyu Kwon[1,2†], Yoon Jae Lee[1,2†], Suyeong Chung[2,3†], Jimin Lee[2,4], Yewon Na[2,4], Youngjin Kwon[2,4], Beomjune Shin[2,4], Allison Bateman[2,4], Jaeho Lee[2,4], Matthew Guess[2,4], Jung Woo Sohn[5*], Jinwoo Lee[6*], Woon-Hong Yeo[2,4,7*]

[1]School of Electrical and Computer Engineering, Georgia Institute of Technology, Atlanta, GA 30332, USA.
[2]Center for Wearable Intelligent Systems and Healthcare, Georgia Institute of Technology, Atlanta, GA, 30332, USA
[3]Department of Aeronautics, Department of Mechanical and Electronic Convergence Engineering, Kumoh National Institute of Technology, Gumi, 39177, Republic of Korea
[4]George W. Woodruff School of Mechanical Engineering, Georgia Institute of Technology, Atlanta, GA 30332, USA.
[5] School of Mechanical System Engineering, Kumoh National Institute of Technology, Gumi, 39177, Republic of Korea
[6] Department of Mechanical, Robotics, and Energy Engineering, Dongguk University, Seoul, 04620, Republic of Korea.
[7] Wallace H. Coulter Department of Biomedical Engineering, Parker H. Petit Institute for Bioengineering and Biosciences, Neural Engineering Center, Institute for Materials, Institute for Robotics and Intelligent Machines, Georgia Institute of Technology, Atlanta, GA, 30332 USA
† K. Kwon, Y. J. Lee, and S. Chung equally contributed to this work

*Corresponding author. Email: Prof. Woon-Hong Yeo (whyeo@gatech.edu), Prof. Jinwoo Lee (jlee484@dgu.ac.kr), and Prof. Jung Woo Sohn (jwsohn@kumoh.ac.kr).

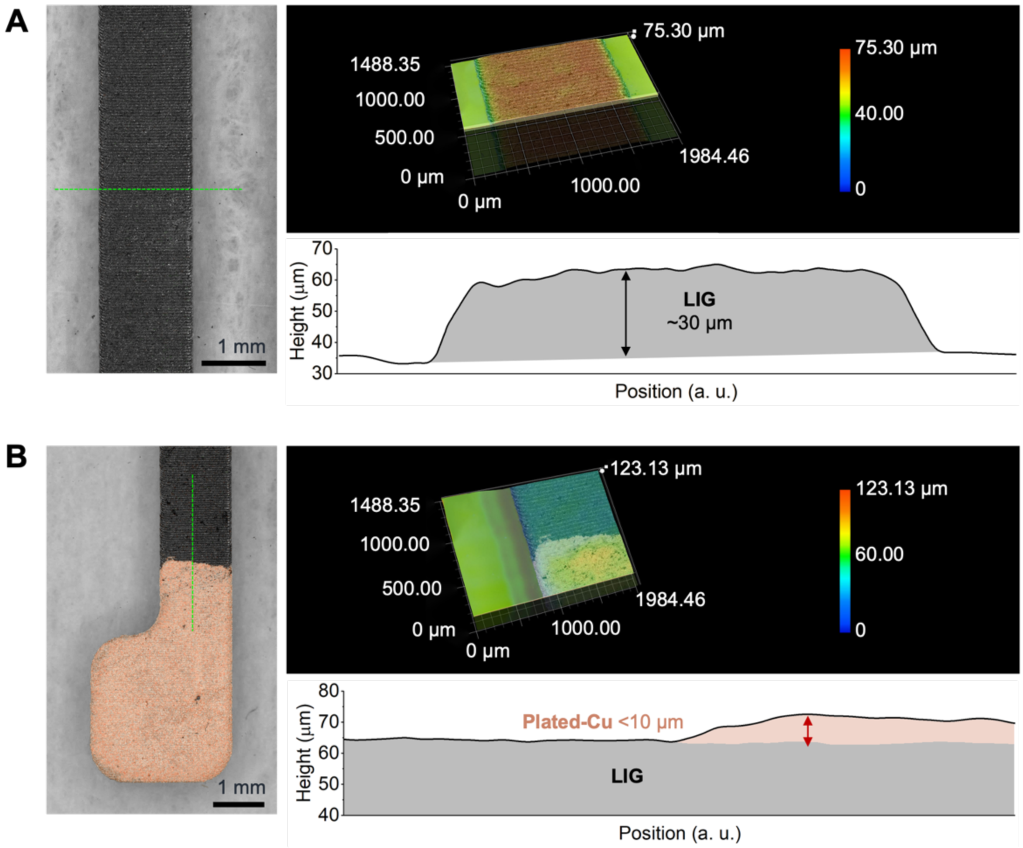

Figure S1. Height profile of LIG electrode. (A) LIG electrode. (B) A portion of LIG is covered by plated copper (Cu).

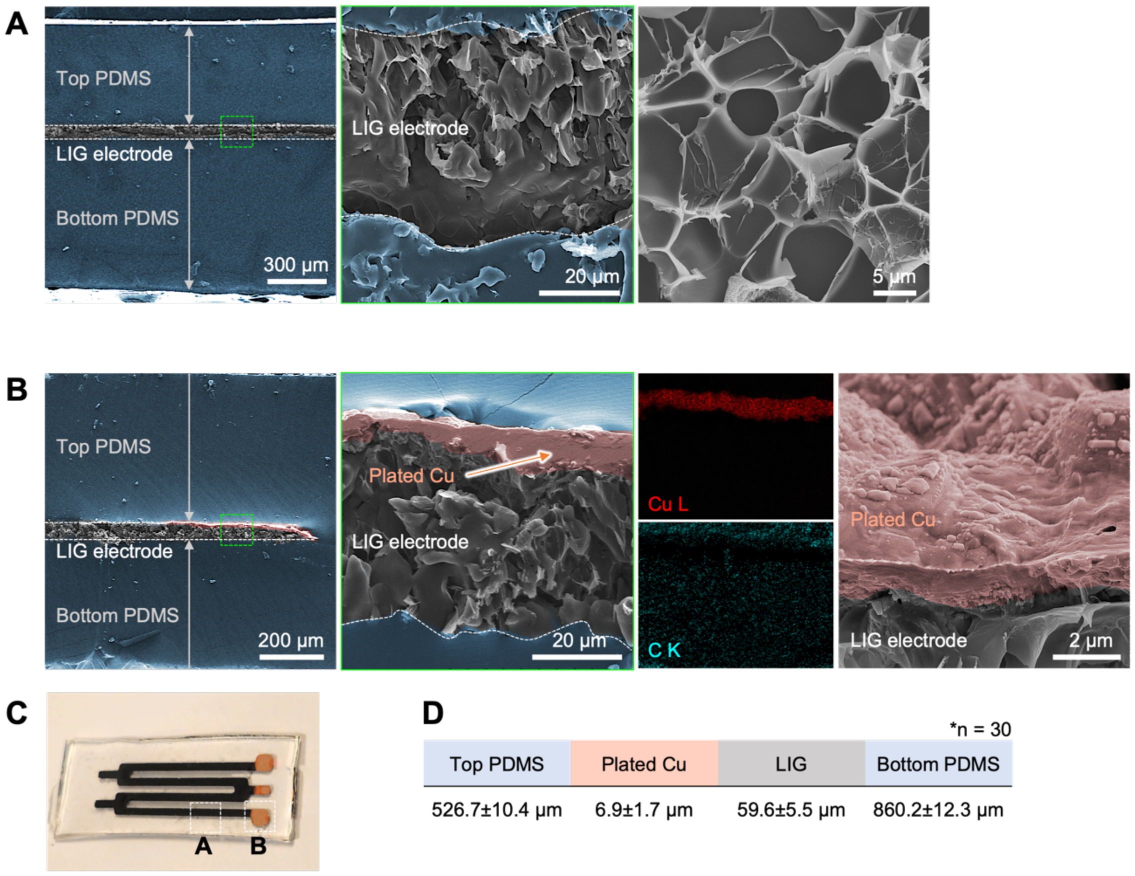

Figure S2. Microstructure of LIG electrode. Cross-sectional and top views of (A) LIG electrode, and (B) a portion of LIG covered by plated copper (Cu) accompanied by elemental mapping results for Cu and carbon. (C) Photograph of LIG electrode. (D) Thickness information for each layer, including top PDMS, plated Cu, LIG, and bottom PDMS layers.

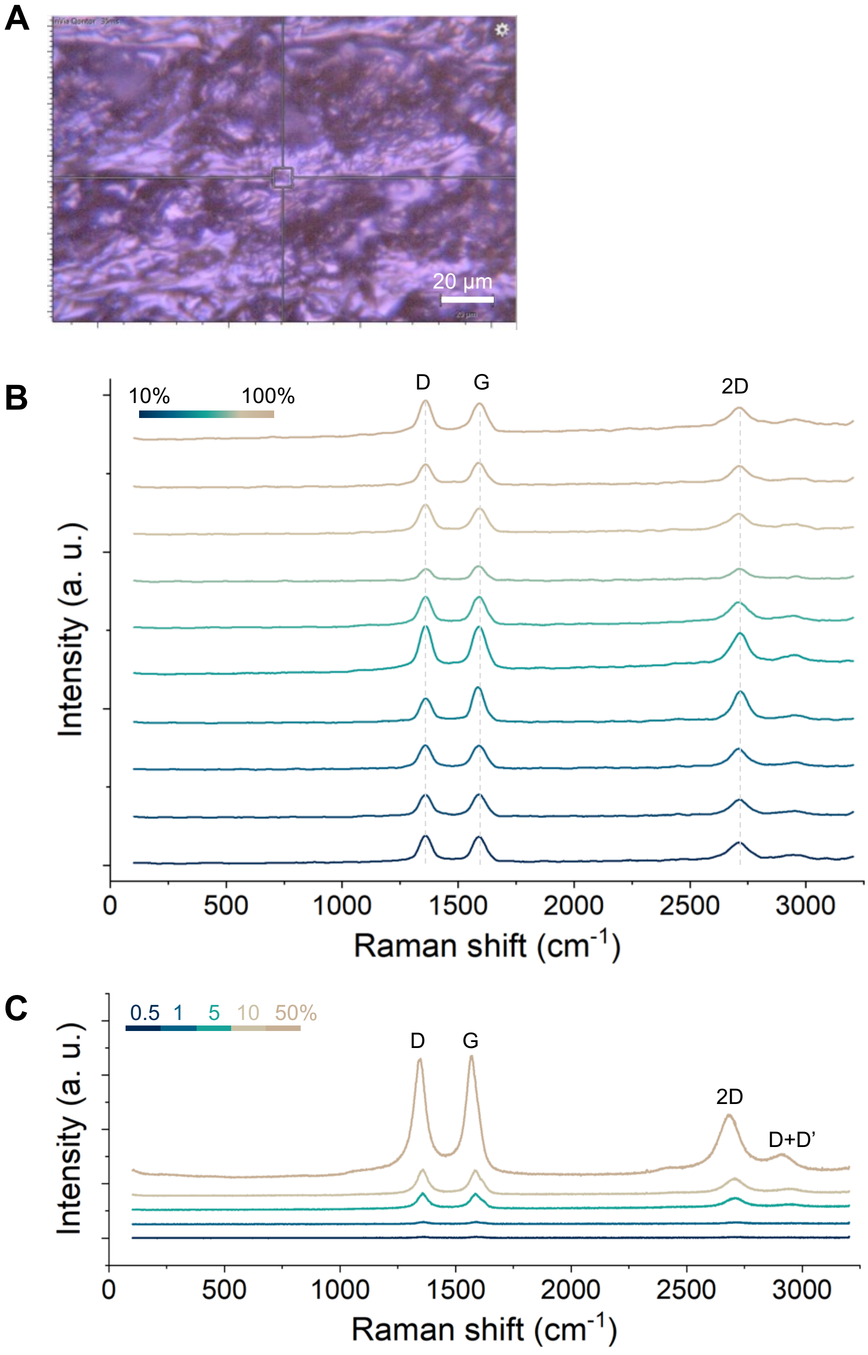

Figure S3. Raman spectra of LIG. (A) Top-view micrograph of LIG pattern; (B) Raman spectra of LIG, patterned with varying laser power intensities (12.4 W) during the laser scribing process; C Impact of Raman laser intensity (7.5 W) on the spectral characteristics of LIG. With 7.5 W of laser power applied, a distinct peak at approximately 2900 cm^{-1} was observed, indicative of the D+D band.

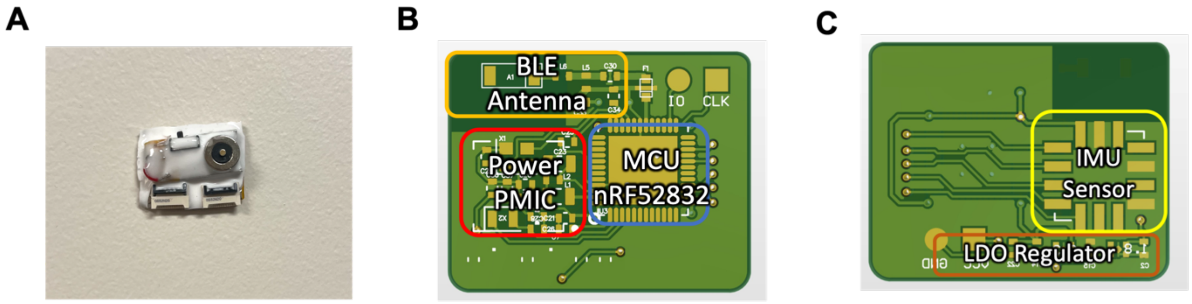

Figure S4. Wearable IMU sensor. (A) Top-view of wearable IMU sensor; (B) Top view and (C) Bottom view of the flexible circuit with the labeled IC components.

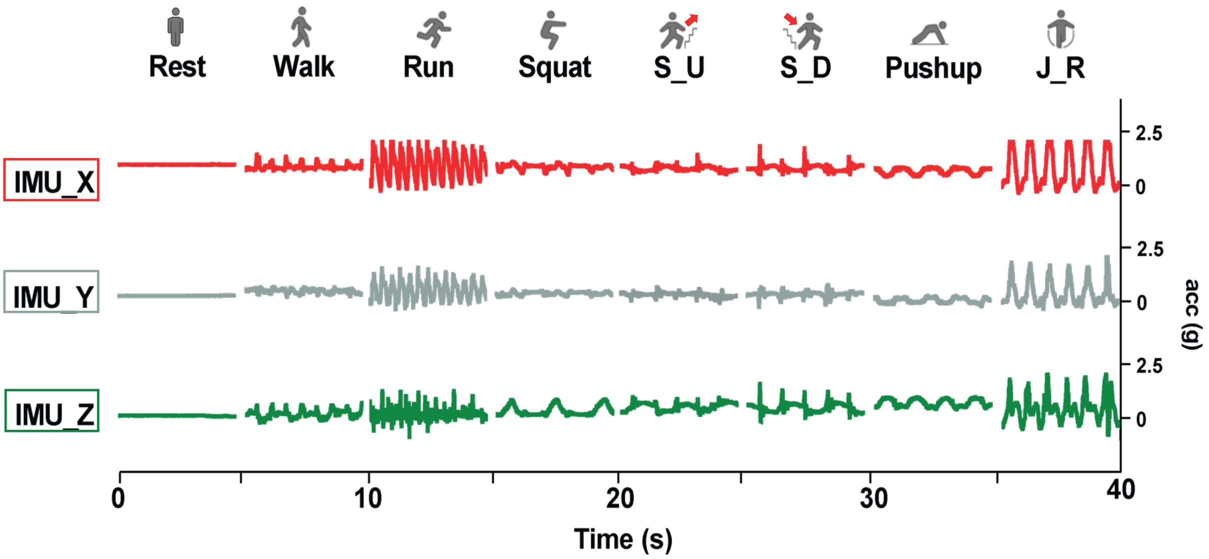

Figure S5. IMU pattern of all joint movements for each workout.

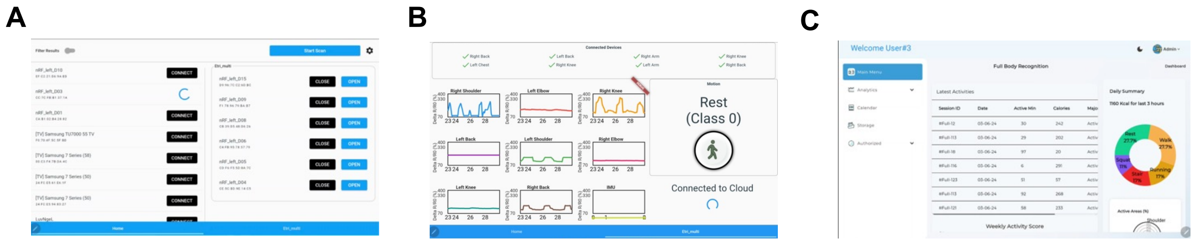

Figure S6. Screenshots of the cloud-based Android GUI that can (A) connect wireless sensors, (B) conduct real-time signal recording and full-body movement classification, and (C) generate a comprehensive report.

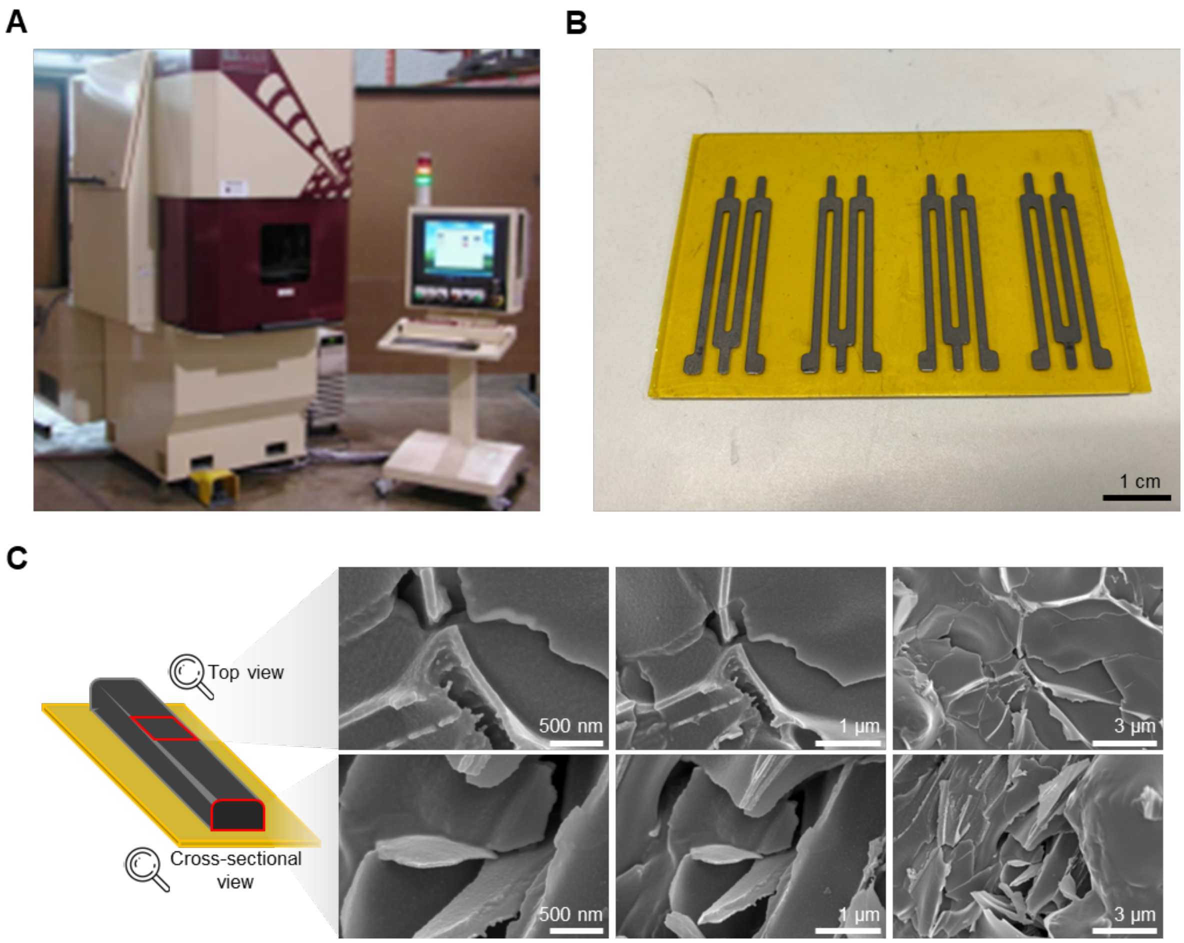

Figure S7. Laser printing setup for the LIG electrodes. (A) Alabama UV Laser machine for LIG electrode printing; (B) The sample of LIG electrode under UV laser printing on PI substrate. (C) High-magnified FE-SEM micrographs of the LIG electrode: top view (top row) and cross-sectional view (bottom row)

A B C

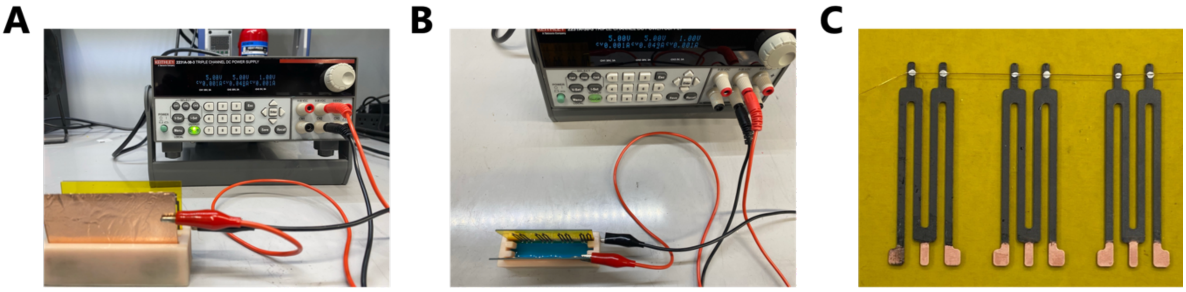

Figure S8. Electroplating setup for the interconnector of LIG electrodes. (A) Front view of the electroplating system for doping Cu on LIG electrode; (B) Side view of the electroplating system; (C) The result of the Cu-doped LIG electrode after electroplating.

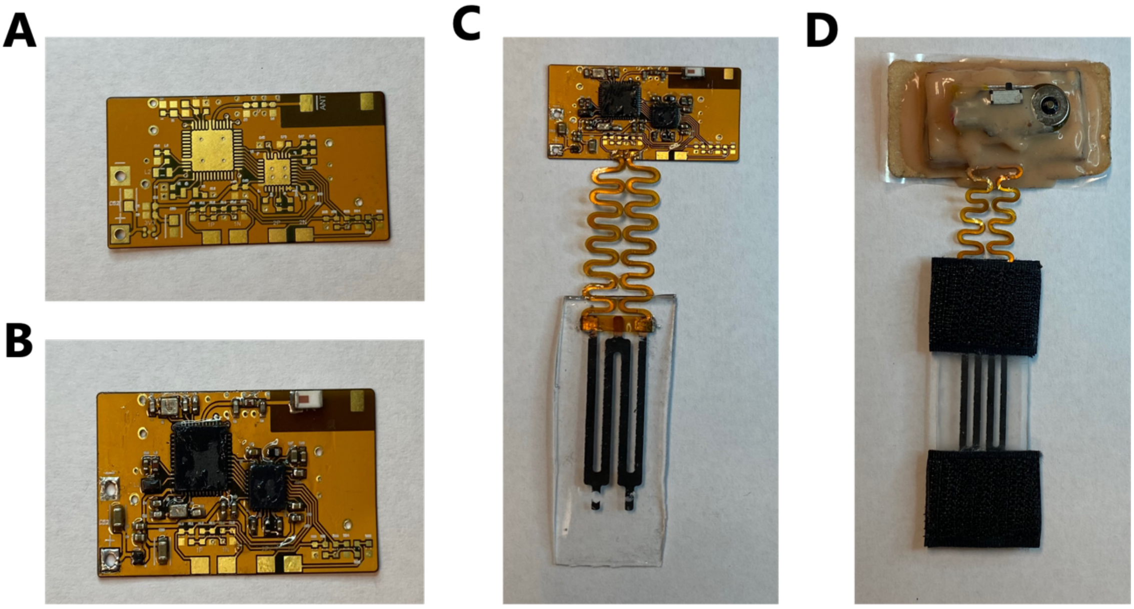

Figure S9. Wearable strain sensor fabrication process. (A) Bare fPCB board; (B) Reflow soldered chips integrated on the fPCB with firmware programmed microcontroller; (C) LIG electrodes soldered to the fPCB along with the battery to power up the entire circuit; (D) Taxtile-integrated LIG electrode using Velcro tape and fully integration encapsulated with the silbione layer.

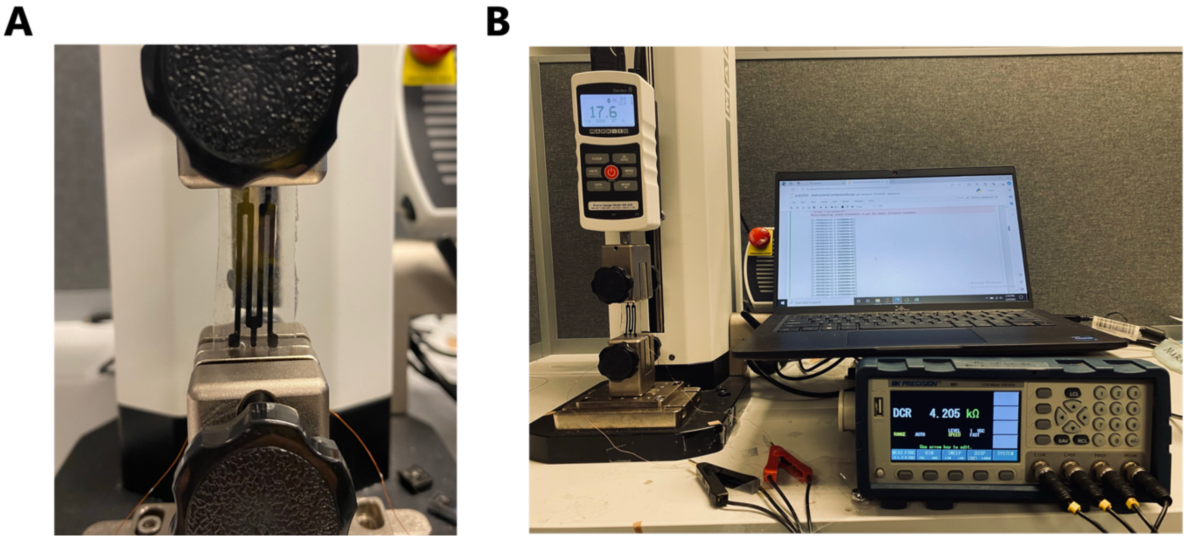

Figure S10. Mechanical test setup for the LIG strain sensor electrodes. (A) LIG electrode under Mark-10; (B) Comprehensive data acquisition system configuration for Mark-10, featuring an LCR meter integration for continuous BK precision measurements via a Python program.

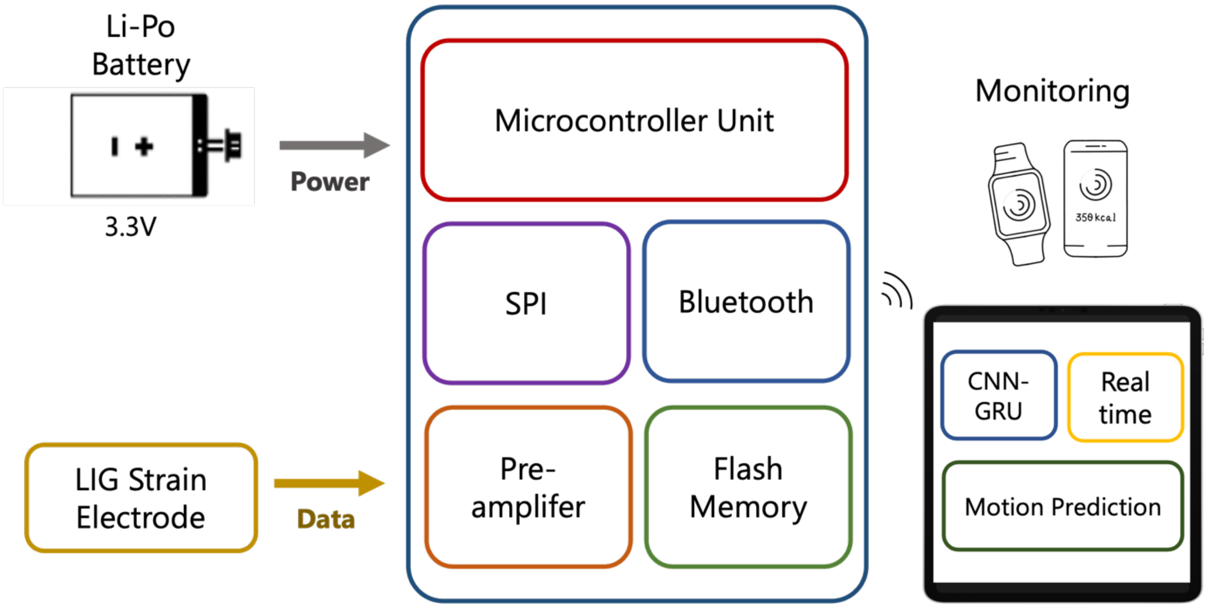

Figure S11. Schematic illustration of the device on power, hardware structure, data, and the real-time motion monitoring system.

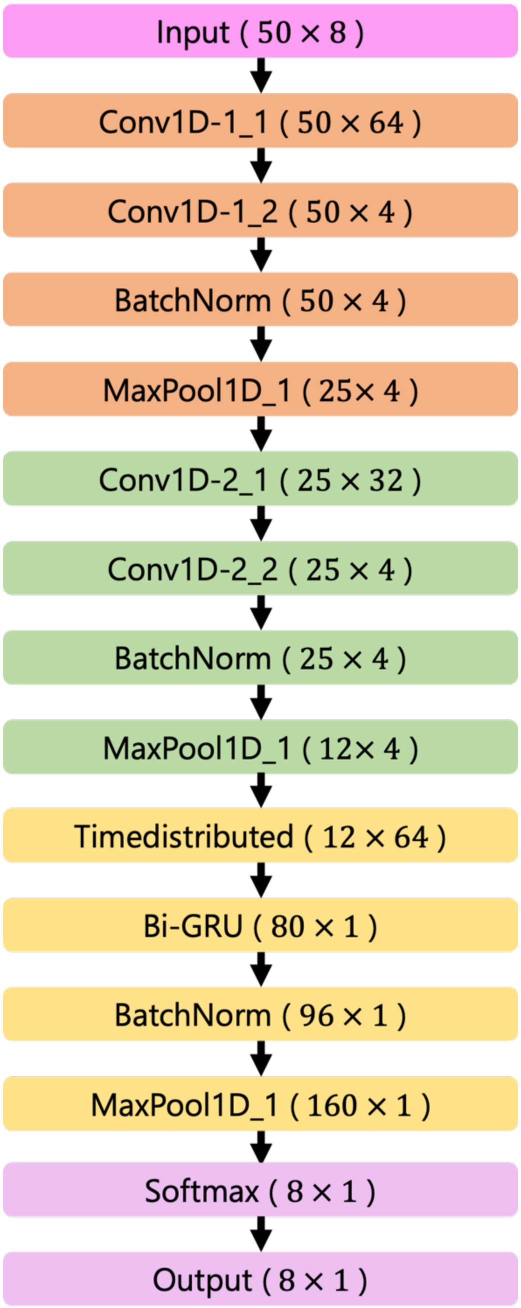

Figure S12. Machine learning architecture for full-body motion classification. The architecture of the proposed CNN-GRU model for full-body classification

Table S1. Machine learning layer information for full-body movement classification. Detailed information about the layers of the proposed network for full-body movement classification.

Layer	Output	# filter	Kernel size	Option
Input	50×8			
Conv1D-1_1	50×64	64	3	Padding = Same<br>Activation = PReLU
Conv1D-1_2	50×4	4	4	Padding = Same<br>Activation = PReLU
BatchNorm	50×4			
MaxPool1D-1	25×4		4	
Conv1D-2_1	25×32	32	5	Padding = Same<br>Activation = PRelu
Conv1D-2_1	25×4	4	4	Padding = Same<br>Activation = PReLU
BatchNorm	25×4			
MaxPool1D-2	12×4		4	
Timedistributed	12×64	64		
Bi-GRU	80	40		Return_sequences = False<br>Activation = tanh<br>$\rho = 0.10$
Flatten-1	96			Unit = 96
Flatten-2	160			Unit = 160
Dropout	160			$\rho = 0.20$
Flatten_final	8			Activation = softmax
Output	8			
